# Signatures of the Venezuelan Humanitarian Crisis in the First Wave of COVID-19: Fuel Shortages and Border Migration

**DOI:** 10.3390/vaccines9070719

**Published:** 2021-07-01

**Authors:** Margarita Lampo, Juan V. Hernández-Villena, Jaime Cascante, María F. Vincenti-González, David A. Forero-Peña, Maikell J. Segovia, Katie Hampson, Julio Castro, Maria Eugenia Grillet

**Affiliations:** 1 Academia de Ciencias Físicas, Matemáticas y Naturales, Palacio de las Academias, Av. Universidad, Caracas 1030, Venezuela; mariaeugenia.grillet@gmail.com; 2 Laboratorio de Biología de Vectores y Parásitos, Instituto de Zoología y Ecología Tropical, Facultad de Ciencias, Universidad Central de Venezuela, Caracas 1058, Venezuela; juanv.hernandezv@gmail.com; 3 Grupo de Biología Matemática y Computacional, Departamento de Ingeniería Biomédica, Universidad de Los Andes, Bogotá 111711, Colombia; je.cascante10@uniandes.edu.co; 4 Department of Medical Microbiology, University Medical Center Groningen, University of Groningen, 9713 GZ Groningen, The Netherlands; mfvg87@gmail.com; 5 Biomedical Research and Therapeutic Vaccines Institute, Ciudad Bolívar 8001, Venezuela; davidnica1991@hotmail.com; 6 Instituto de Medicina Tropical, Facultad de Medicina, Universidad Central de Venezuela, Caracas 1058, Venezuela; segoviamaikell@gmail.com (M.J.S.); juliocastrom@gmail.com (J.C.); 7 Institute of Biodiversity, Animal Health and Comparative Medicine, University of Glasgow, Glasgow G12 8QQ, UK; Katie.Hampson@glasgow.ac.uk

**Keywords:** SEI models, metapopulations, Venezuela, SARS-CoV-2, drivers of transmission, spatial incidence

## Abstract

Testing and isolation have been crucial for controlling the COVID-19 pandemic. Venezuela has one of the weakest testing infrastructures in Latin America and the low number of reported cases in the country has been attributed to substantial underreporting. However, the Venezuelan epidemic seems to have lagged behind other countries in the region, with most cases occurring within the capital region and four border states. Here, we describe the spatial epidemiology of COVID-19 in Venezuela and its relation to the population mobility, migration patterns, non-pharmaceutical interventions and fuel availability that impact population movement. Using a metapopulation model of SARS-CoV-2 transmission dynamics, we explore how movement patterns could have driven the observed distribution of cases. Low within-country connectivity most likely delayed the onset of the epidemic in most states, except for those bordering Colombia and Brazil, where high immigration seeded outbreaks. NPIs slowed early epidemic growth and subsequent fuel shortages appeared to be responsible for limiting the spread of COVID-19 across the country.

## 1. Introduction

The incidence of reported COVID-19 cases in Venezuela is one of the lowest in Latin America. As of 17 May 2021, a total of 216,415 cases (0.9% of the population) and 2411 deaths have been officially confirmed. Testing, however, has also been among the lowest in the region [1,2]. An average of 17 PCR tests per 1000 inhabitants had been carried out as of 21 December 2020 [3] (accessed on 25 May 2021). Except for five laboratories approved by the government, testing initiatives have been banned [1]. Positivity rates from 25–50% after June 2020 suggest an epidemic that is 5–7 times larger than officially reported. Thus, substantial under-testing casts doubt on the official tally and the country’s capacity for surveillance to interrupt SARS-CoV-2 transmission [2].

**Figure A1:**
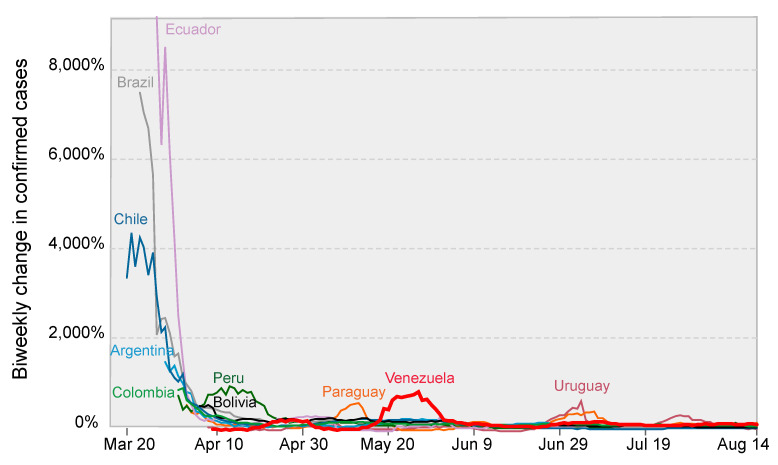
Biweekly changes in confirmed cases of COVID-19 per million people in South American countries. The highest biweekly changes indicate when the initial growth phase occurred in each country. Venezuela lagged behind most countries in the region. Figure adapted from Our World Data [32] (accessed on 30 May 2021).

Despite the significant underestimation of case incidence, epidemic growth in Venezuela appeared to lag behind other countries in the region (Figure A1). Pre-existing air-traffic restrictions and a complete country lockdown shortly after first detection of SARS-CoV-2 limited the number of initially imported cases. In addition, a military-enforced quarantine reduced population mobility during the early stages of the epidemic. Although compliance with restrictions rapidly decreased, as 45% of the active population works in the informal economy and depends on a daily income [4] (accessed on 25 May 2021), a severe shortage of gasoline during the first eleven weeks of the epidemic inevitably reduced people’s movement, particularly between states.

In contrast to this reduced mobility within the country, movement across national borders increased substantially during the first few months of the pandemic. Thousands of Venezuelan residents who had recently fled the ongoing humanitarian crisis in the country were forced to return as the pandemic hit the region’s economy [5]. Between 13 March and 21 May, 40,000–80,000 documented migrants entered the country through humanitarian missions, mainly from Colombia, Brazil, Ecuador and Perú [6], in addition to unrecorded informal crossings through four border states [7]. Soon after, cases were detected in four border states and incidence rapidly increased. From mid-June 2020 on, the Venezuelan government issued seven-day shelter-in-place orders every 15 days to reduce population movements. Compounding this, from mid-August to early October 2020, gasoline availability dropped to below 9% of pre-pandemic levels.

Our aim was to understand how the humanitarian crisis in Venezuela affected the spread of COVID-19. As such, we investigate the spatial dynamics of COVID-19 in Venezuela, specifically in relation to population mobility, migration, non-pharmaceutical interventions (NPIs) and fuel availability during the first wave of the epidemic (13 March–21 December 2020). Using a susceptible-exposed-infectious (SEI) metapopulation model, we explore mobility patterns and their relationship with the spatial distribution of observed cases. We further analyzed the effect of mobility restrictions (shelter-in-place orders) and fuel shortages to identify the major drivers of the Venezuelan epidemic.

## 2. Materials and Methods

### 2.1. Spatial Epidemiology and Modeling

**Figure 1:**
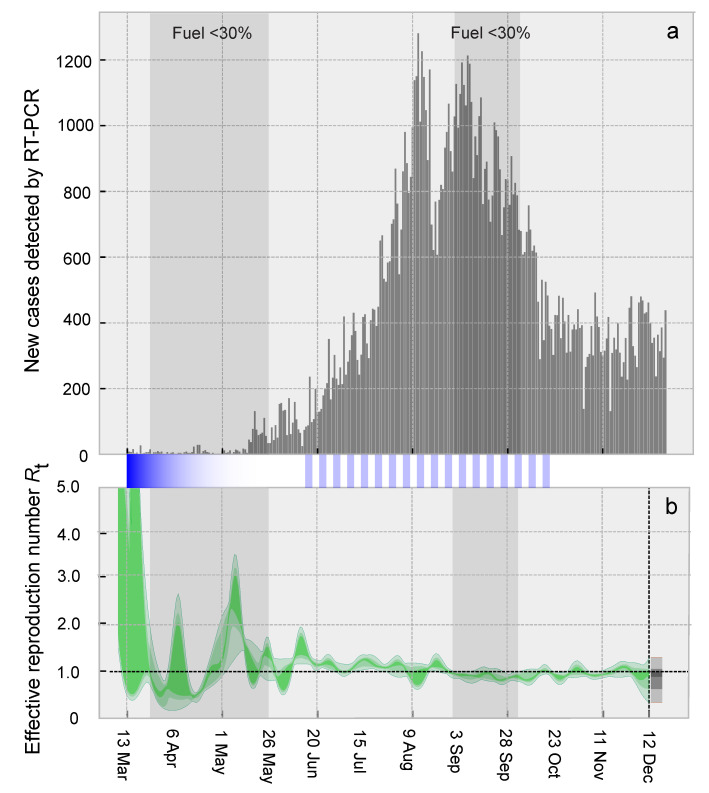
(**a**) Official record of daily infections in Venezuela detected by RT-PCR between 13 March and 21 December and (**b**) effective reproductive numbers estimated (green) and projected (gray) from incidence data adjusted for positivity (Figure A2), with a seven-day sliding window using the EpiNow2 package [9]. Dark, intermediate and light colored curves correspond to 50%, 90% and 95% percent confidence intervals. Dark grey windows show the periods during which less than 30% of stations had no gasoline. Blue bands indicate when stay-at-home orders were in place, and the color transparency the approximate level of compliance.

**Figure A2:**
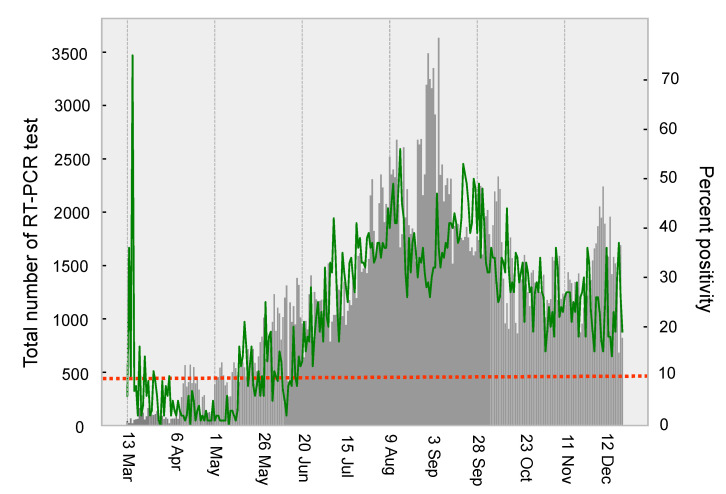
RT-PCR testing in Venezuela. The grey bars indicate the total number of tests carried out and the green line estimates the percent positivity. The red dotted line corresponds to the positivity recommended by the WHO for an adequate case tracing.

Data from Venezuela were obtained from official reports and corrected using the percent positivity to account for substantial underreporting (Figure A2) [8]. To explore the geographical spread of SARS-CoV-2 across Venezuela in 2020, we generated a heatmap of daily cases between 13 March and 21 December across the country’s 25 states. Maps were created in the Q-GIS software (version 2.18.9-Las Palmas de G.C., GNU-General Public License, https://www.qgis.org/es/site/ (accessed on 25 June 2021)) and the heatmap was constructed in the ggplot package from R free-software (The R-Development Core Team, http://www.r-project.org (accessed on 25 June 2021). We further estimated the instantaneous reproductive numbers, $R_{t}$, from incidence data with a seven day sliding window using the EpiNow2 package [9] for R (Figure 1b).

To infer SARS-CoV-2 dynamics, we adapted a metapopulation model with a susceptible- exposed-infectious (SEI) compartmental structure that accounted for undocumented infections (Appendix A.1) [10]. The trajectories of susceptible, $S_{i}$, exposed, $E_{i}$, and documented infections $I_{i}$ and undocumented infections $U_{i}$ were estimated from within- and between-state transmission (Equations (A1)–(A5)). We assumed that susceptible, documented or undocumented infectious individuals moved between states or from neighboring countries, with the daily number of people moving dependent on the distance between state centroids and their population sizes, according to a gravity function [11] (Equation (A6)). The sustained immigration of individuals from neighboring Colombia and Brazil was modeled by including two additional subpopulations, and occurred in only one direction, from Colombia or Brazil to the border states: Amazonas, Apure, Bolívar, Táchira and Zulia. Migrants entering these border states travelled from different provinces or states in Colombia or Brazil. We therefore assumed that contact rates with infected individuals were proportional to the fraction of the total population infected in each of these two countries in their border states. We also assumed that mixing within states and transmission across states was homogeneous. We used a mean delay of seven days between infection and reporting for Venezuela case data [12] (accessed on 25 May 2021). Incidence data for Brazil and Colombia were obtained from the John Hopkins University repository (coronavirus.jhu.edu/map.html (accessed on 25 June 2021)) and were modelled with no delay.

### 2.2. Model Parametrization and Initialization

We used an average duration of latency, *Z*, and infection, *D*, estimated from serial intervals of COVID-19 [13]. The reporting rate, α, was approximated by comparing officially reported case numbers with those inferred from the first three reported deaths, using a branching process for forecasting new cases [14]. The starting scenario for Venezuela consisted of an initial seed of documented $I_{i}$ and undocumented cases $U_{i}$ estimated according to the first reported deaths, assuming a basic reproductive rate ($R_{o}$) of 2.4 and a case fatality rate (CFR) of 3% (Table 1). For Colombia and Brazil, we used seeds that would reproduce reported incidence, assuming 50% reporting rates in these countries.

**Figure A3:**
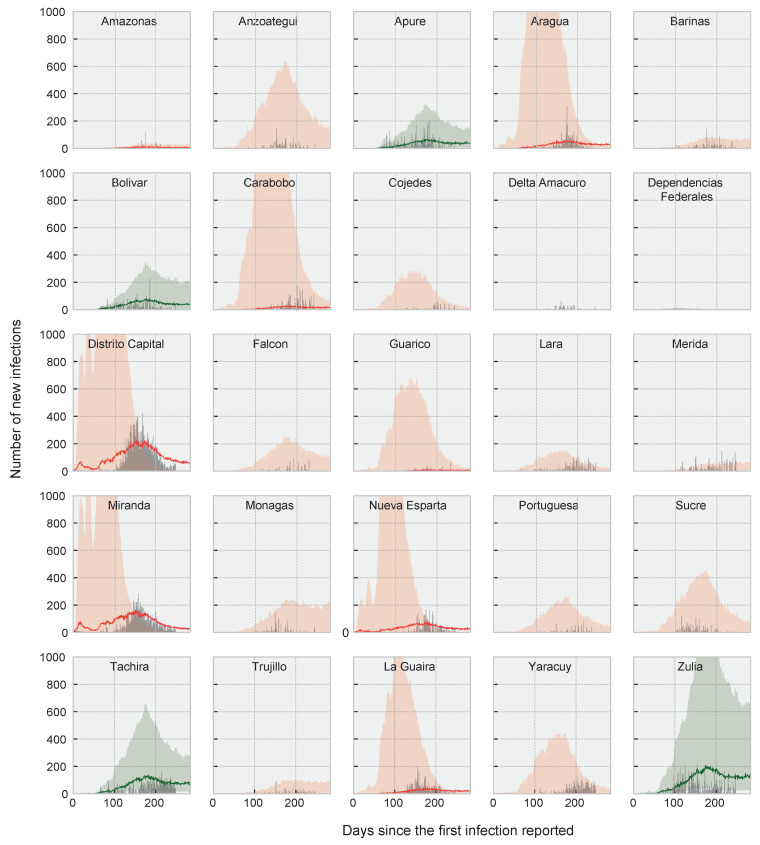
Daily infections with SARS-CoV-2 in Venezuela during the first 285 days. Gray bars are the number of cases confirmed with RT-PCR and reported by health authorities. The lines and shades are the mean and 50% confidence intervals of daily infections documented in non-border (red) and border (green) states predicted by the SEI metapopulation model, assuming that only 25% of all cases are documented.

To explore values of daily travelers that would reproduce the observed local epidemics, we investigated a range of daily movement rates by modifying the proportionality constant, θ, the maximum number of daily travelers between any two states within Venezuela (Table 1), of a gravity function scaled to [0, 1] (Equation (A6)). Changes in θ modify the absolute values for daily movements between all states, while leaving their relative values unchanged. The effect of migration from neighboring countries was investigated by calibrating the number of travelers entering Venezuelan border states from Colombia and Brazil (Table 1), to generate the observed infection incidence in border states, given transmission rates similar to the rest of the country (Figure A3).

### 2.3. Identifying the Drivers of Contagion

We explored the effects of NPIs and gasoline availability on population mobility and contagion rates. Interventions in Venezuela included shelter-in-home orders, closure of parks and public offices and prohibition of commercial or social activities in addition to a substantial reduction of domestic air and terrestrial traffic. A 7 × 7 intermittent scheme consisting of seven-day lockdowns every 15 days was implemented from mid-June to mid-November during 2020. The residence time from Google Mobility was used as an indicator of population mobility and measures the excess hours spent at home (sleeping location), relative to that time spent at home before COVID-19 restrictions. As a proxy for gasoline availability, we estimated the fraction of gas stations open in a given week from country-wide surveillance [15].

A wavelet analysis was performed to investigate the periodicity of the residence time and the reproductive number using Wavelet Test for MATLAB [5]. This package uses a Morlet wavelet base function to identify the frequency and location of periodic components. We expected to find a transient periodic signal with a 15-day cycle from mid-June to mid-November, when the lockdown/relaxation scheme was implemented across the country.

Changes in mobility can be driven by lockdown/relaxation switches, or by longer term responses not associated with these NPIs. We therefore decomposed the residence time into its trend and periodical signal using moving averages estimated by local polynomial regression fitting (stl package in R). We assumed that the periodic component resulted from the variation in mobility associated with compliance to NPIs, while the trend describes longer term variation independent of NPIs. By comparing regression models using these two variables, we assessed the relative contribution of the bi-weekly interventions and any longer-term changes in residence time to the instantaneous reproductive number $R_{t}$.

## 3. Results

The official record describes the epidemic curve in Venezuela initially at low incidence between March and mid-May 2020, followed by a steady increase from 15 May onwards, which levelled off from mid-August 2020. Declines in case incidence were evident from late September until the end of 2020 (Figure 1a).

### 3.1. Low Connectivity, Migrants and the Development of the Epidemic

**Figure 2:**
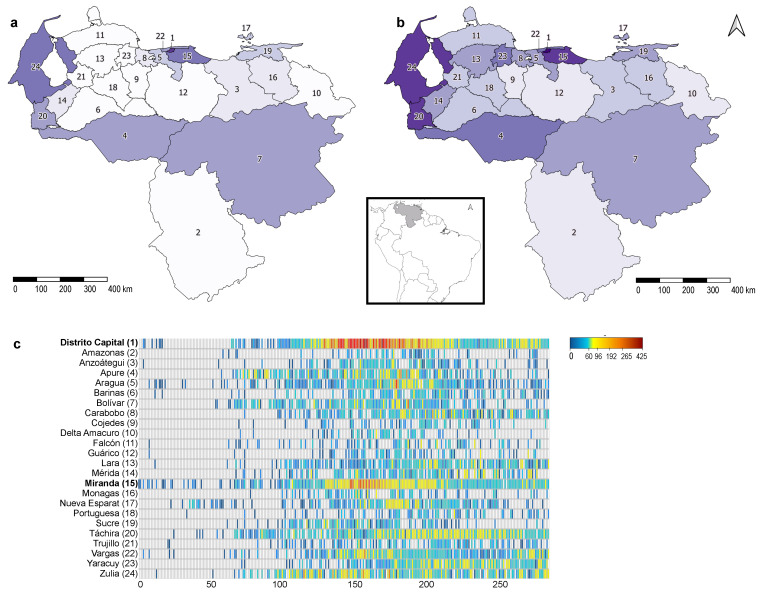
COVID-19 cumulative cases (incidence) per state in Venezuela from March to August (**a**) and December (**b**) 2020. Heatmap of daily reported infections (legend color bar) across states (**c**). State numbers in the map correspond to state names in the heatmap.

The first wave of the COVID-19 epidemic showed substantial geographical heterogeneity (Figure 2). The epidemic began in the capital region (Miranda, Distrito Capital, and Vargas states) in the north-central part of the country, where the first cases from Europe were documented (Figure 2a). New infections were recorded in these states almost every day after the first two months, when case reporting was no longer intermittent (Figure 2c). In May, introductions from Colombia and Brazil initiated local epidemics within their bordering states (Apure, Bolívar, Táchira, and Zulia) (Figure 2b). Since then, reported incidence has been significant and sustained in these states, particularly in Zulia (Figure 2a,c). In the Northcentral and Northwest regions (Aragua, Anzoátegui, Carabobo, Cojedes, Portuguesa, and Falcón states), the epidemic onset occurred during July and August 2020 (Figure 2b,c).

**Figure 3:**
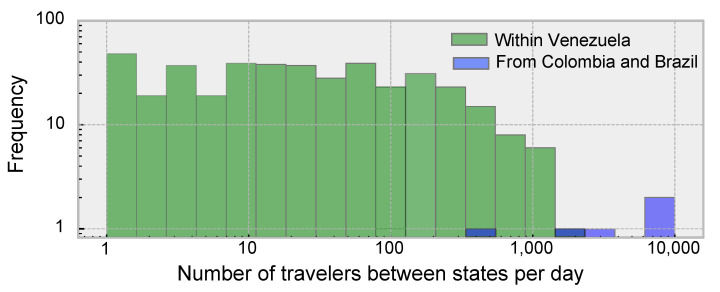
Frequency of daily movements between states used in the simulations with the SEI metapopulation model, derived from the Gravity function and calibrated against observed dynamics. The volume of daily movements within Venezuela was orders of magnitude smaller than from Colombia and Brazil.

In states where the epidemic initially started (Distrito Capital, Miranda), model simulations seeded as described in Table 1 with a reporting fraction of 0.15, produced case incidence curves similar to those officially reported (Figure A3). This suggests that by the time the first case was reported, nearly 600 persons had already been infected in the country, both symptomatic and asymptomatic. Assuming a maximum of 3000 travelers in the most traversed route, modelled epidemics in nearby states (Aragua, Carabobo, Guárico and La Guaira) began around 100 days after the first case was reported in the country, consistent with the observed cases. The frequency distribution of daily travelers between states obtained from the gravity function indicated that less than 100 persons would be traveling daily between most states (Figure 3). In contrast, between 800 and 10,000 travelers per day were required to reproduce epidemic curves similar to those observed in border states. This suggests that a combination of low connectivity within the country and a high influx of migrants from neighboring states would reproduce an epidemic characterized by the delayed start and few highly localized foci during the first 284 days.

### 3.2. NPIs, Gasoline, Mobility and Contagion

**Figure 4:**
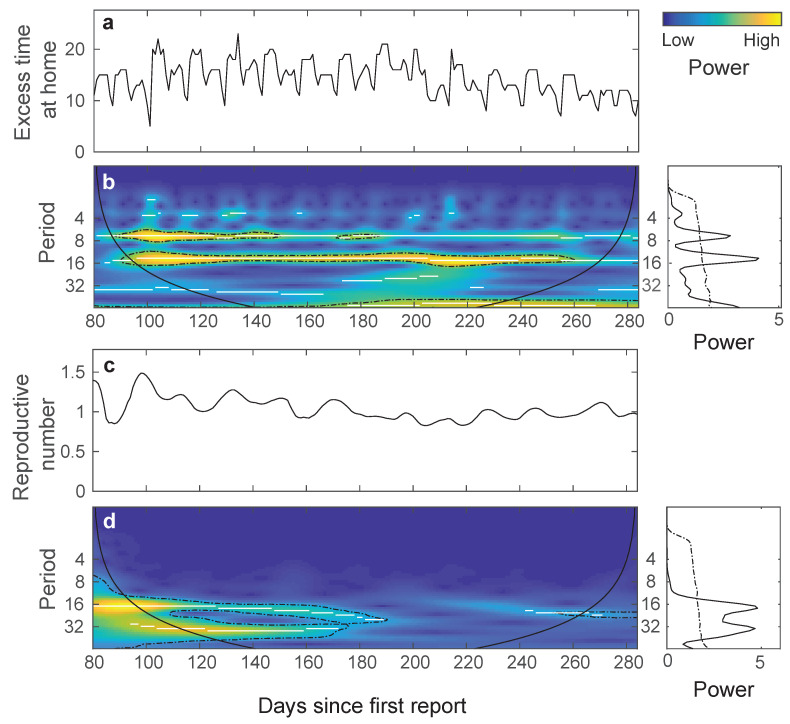
Time series and wavelet power spectra of the residence time (**a**,**b**) and the effective reproduction number (**c**,**d**) between mid-June and mid-December 2020. The colors for power values range from dark blue (low values) to orange (high values). Strong signals with periods of 7 and 15 days are observed in the excess residence time (**b**). A weak and interrupted signal with a period of 15 days is shown for the effective reproductive number (**d**). The black lines correspond to the maxima of the undulations of the wavelet power spectrum. Right: the two global spectra corresponding to each analysis.

The wavelet power spectrum of the residence time revealed high-power signals with periods of 7 and 15 days between mid-June and mid-November 2020 when the 7 × 7 shelter-in-place scheme was implemented (Figure 4a). The 7-day period originated from a lower excess time at home during weekends compared to weekdays, whereas the 15-day period coincided with the seven-day lock-down every 15 days (Figure 4b). In contrast, a weaker and interrupted signal with a 15-day period in the reproductive number suggests that changes in mobility from the NPIs translated weakly into variation in contagion.

Comparison between regression models indicated that excess time at home was a weaker explanatory variable for the reproductive number than its trend component. A lower root mean square error for the trend component (RMSE = 0.1786) compared to the original variable (RMSE = 0.1944) indicates that the performance of the regression model improved when the periodic component was removed from the time series. This suggests that variations observed in $R_{t}$ between mid-June and mid-December were more likely driven by longer term variation in mobility not associated with NPIs. We therefore used the trend component of the residence time to explore the effects of gasoline shortages on $R_{t}$.

**Figure 5:**
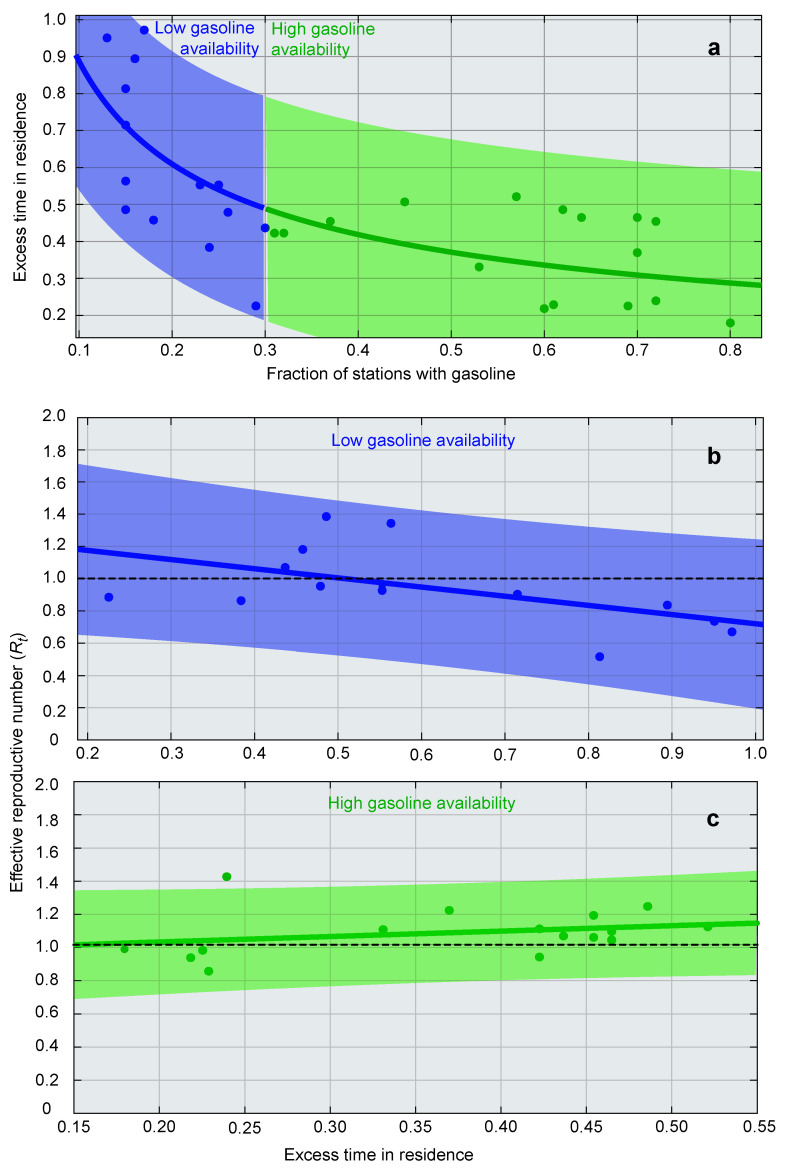
Drivers of contagion in Venezuela. (**a**) Excess time spent at home relative to pre-pandemic (Google Mobility) decreases with the availability of gasoline according to $y=0.2536*x^{-0.5487}$. This dependence was stronger when gasoline availability dropped below 30% (orange area), than when it was above this threshold (green area). (**b**) When gasoline availability decreased below 30%, the effective reproductive number decreased significantly with the excess time spent at home ($R^{2}=0.29$, $F_{\left(1,13\right)}=4.91$, p=0.046). (**c**) When gasoline was available in more than 30% of stations, the effective reproductive number no longer showed a significant relationship with excess time spent at home ($R^{2}=0.07$, $F_{\left(1,13\right)}=1.12$, p=0.278). Two outliers with $R_{t}>2$ were omitted from the analysis.

The trend component in residence time decreased significantly as gasoline became available in a greater fraction of stations (Figure 5a). A better fit to a power function compared to a linear function indicated that this effect was stronger when gasoline availability dropped below 30%. During these periods of severe shortages (<30%), $R_{t}$ decreased with the excess time spent at home (Figure 5b), whereas when gasoline availablity increased (>30%), $R_{t}$ no longer showed a significant association with excess time spent at home (Figure 5c). This suggests that the availability of gasoline was likely a major driver of contagion, reducing $R_{t}$ significantly during periods of severe shortages.

## 4. Discussion

Isolation of symptomatic and asymptomatic persons and restrictions on population mobility have been critical for controlling the COVID-19 pandemic. In Venezuela, where contact tracing has been poor due to limited testing capacity, national cyclic lockdowns were adopted by the government as the main NPI for interrupting transmission. Despite low compliance to these national stay-at-home orders, the epidemic in Venezuela was substantially delayed, compared to other countries in the region. Although the substantial underreporting of infections and deaths indicate that a largely unobserved COVID-19 epidemic occurred in Venezuela, we show that severe shortages of gasoline delayed the initial within-country spread of SARS-CoV-2, mitigating its impact during the first epidemic wave.

In many countries, COVID-19 initially spread from focal epicenters into neighboring regions, generating a gradual decrease of cases with distance [16,17,18]. Our model simulations of spatial spread indicated that the fragmented distribution of observed cases in Venezuela, localized mainly in Distrito Capital and Miranda, where the outbreak began, and in border states, can only be reproduced if the mobility between states is orders of magnitude below that across the country’s borders. Thus, low connectivity within the country combined with high permeability of borders likely resulted in a delayed epidemic in most states, except for those adjoining Colombia and Brazil.

Although statistics on absolute mobility within Venezuela are lacking, several factors are thought to have substantially reduced mobility, including paralysis of ground transportation due to fuel shortages and an absence of vehicle replacement parts [19] (accessed on 25 May 2021), cancellation of domestic flights [20] (accessed on 25 May 2021), and the general economic impact of a three-digit annual inflation [21] (accessed on 25 May 2021). As a result, cities and towns within Venezuela remained relatively disconnected during 2020. Moreover, shelter-in-place orders decreed during the first 90 days reduced mobility by 40% (Google Mobility), but compliance halved after the first 30 days. This combination of pre-existing limitations on air and ground transport and high initial compliance to lockdown orders contributed to the slow start of the epidemic in Venezuela.

Later restrictions in mobility decreed by the government, however, appeared to have little effect on contagion. According to Google Mobility, the seven-day stay-at-home orders implemented every 15 days between mid-June and mid-November reduced people’s movements by 5% every two weeks, but these biweekly reductions did not apparently influence $R_{t}$. Mobility represents an important proxy of social distancing [22], but the relaxation of strict control can decouple mobility and transmission [23]. Instead, declines in transmission over longer time-scales were associated with gas stations closures, suggesting that fuel availability was a major factor limiting transmission in Venezuela. Fuel availability dropped below 30% for 60 days between March and April and again for 30 days between September and October of 2020. These periods of severe shortage not only coincided with the lowest transmission rates, but regression models also suggested that longer term variation in mobility, after biweekly variations were removed, better explained the observed changes in $R_{t}$. We therefore conclude that the crippling effect of fuel shortages on ground transportation slowed the spread of SARS-CoV-2 within Venezuela during 2020.

While transit between states was substantially reduced, cross-border movement from Colombia and Brazil increased during the early phase of the pandemic. As of 9 November 2020, about 4.6 million Venezuelan refugees, migrants and asylum seekers were reported by host Latin American and Caribbean countries. Nearly 5000 residents were estimated to emigrate daily through the borders with Colombia and Brazil, to escape the ongoing humanitarian crisis [24]. However, as the pandemic hit the global economy in 2020, migration patterns were reversed [25]. During the 60 days that followed the first detected case, an average of 1000 persons returned daily through humanitarian missions [6] (accessed on 15 May 2021) and countless informal crossings occurred through Apure, Bolívar, Táchira and Zulia, the few states where outbreaks developed early during the epidemic [7]. With population movement concentrated mainly at the borders, the epidemic’s impact in 2020 was mainly localized in the capital region and four border states.

The impact of COVID-19 in Venezuela was expected to be high in light of the ongoing humanitarian crisis [24]. However, only 81 deaths per million have been reported as of 13 May 2021, a tally orders of magnitude less than neighboring Colombia (1616) and Brazil (2046) and only comparable to some African countries. Countries in Africa also appeared to have a delayed first wave [26], with younger age, cross-reactive immunity, previous vaccinations or experience with prior epidemics all invoked as possible causes. However, several studies indicate that insufficient data represent a major explanation [27,28,29], with data on excess mortality from funeral houses or burials demonstrating the significant under-ascertainment of deaths. In Venezuela, official data are hard to come by. However, close monitoring by NGOs of the incidence of fatalities in health personnel indicates that the number of deaths from COVID-19 in this group is 4–5 higher than the total offically reported fatalities (COVID19 NGO Surveillance Initiative, Weekly Bulletin 2 May 2021. Unpublished Data). Thus, concomitant with the slow spread of SARS-CoV-2 due to shortages of gasoline, the significant under-ascertainment of COVID-19 deaths has contributed to an official narrative that the Venezuelan epidemic has been controlled, promoting a false sense of security.

There are a number of limitations to our study, particularly concerning the use of indirect indicators. First, because RT-PCR testing is centralized in Venezuela and the numbers of tests performed in each state are not available, we were only able to estimate the average positivity across the country. Using the average positivity in remote states, where positivity is likely to have been higher because testing is focused on symptomatic patients, will have resulted in an underestimation of the true infection rates, but not the epidemic onset in each state. Second, because mobile services have low penetration in Venezuela (49% in 2019), Google mobility data may not be representative of the population as a whole, but only of sectors of society with higher capacity to comply with restrictions. This means that mobility could be underestimated, but the relative contributions of the periodic and trend components are not expected to change. Third, the use of a gravity function to model movement across states requires previous data on traffic to fit the parameters. However, because data on movement is not available, we had to adjust parameter values intuitively to generate traffic between states consistent with anecdotal information collected from news agencies.

We conclude that signatures of the Venezuelan humanitarian crisis are clearly evident in the first wave of COVID-19, both the delayed onset and limited spread due to movement restrictions and subsequent fuel shortages, and the high incidence in border states from migration. Despite the delayed first wave, a second wave appears to now be progressing more aggressively. Infections and deaths continue to be substantially underreported and health infrastructure is strained. Between January and May 2021, the percentage occupancy of intensive care units in hospitals increased from 20% to 60%, with Miranda, Distrito Capital, Zulia and Anzoátegui nearly all at full capacity (COVID19 NGO Surveillance Initiative, Weekly Bulletin, 18 April 2021, unpublished data). In April 2021, the number of deaths due to respiratory infections reported in hospitals almost doubled that observed during the first peak in August 2020 (COVID-19 NGO Surveillance Initiative, Weekly Bulletin, 18 April 2021, unpublished data). We therefore caution that unless steps are taken to rebuild the failing public health infrastructure, the death toll due to COVID-19 is expected to increase. Moreover, Venezuela is falling behind in vaccination. While concerns are mounting about the rise of more contagious variants [30], the vaccination rate in Venezuela is among the slowest in the region (<1%). Political obstacles have curbed several attempts to deliver vaccines, including those by the Pan American Health Organization (PAHO) [31] (accessed on 13 May 2021). As a result, the country is moving towards a larger and more severe second wave, with few mitigation strategies available, other than enforcing lockdown restrictions. The pandemic has enabled autocratic governments to change laws and introduce restrictions, but public health investment and transparent communication of science underpinning regulations have proven to be crucial factors in minimizimg the impact of this disease in many countries.

## Figures and Tables

**Table 1:** Epidemiological parameters, initial conditions and range of daily movements used for simulations within the metapopulation SEI model.

Parameter	Values
Transmission rate (β, days${}^{-1}$)	$R_{t}/D$
Latency period (*Z*, days)	3.9
Infectious period (*D*)	5
Reporting fraction (α)	0.15
Initial seeds Distrito Capital ($I_{o},U_{o}$)	120, 240
Initial seeds Miranda ($I_{o},U_{o}$)	100, 200
Maximum daily local travelers (θ, days${}^{-1}$)	100–5000
Daily travelers from Colombia	3500–10,000
Daily travelers from Brazil	500–2000

## Appendix A

### Appendix A.1. Delayed Start of First Epidemic Wave

The epidemic growth in Venezuela appeared to have lagged behind other countries in the region. Reporting can vary significantly from day to day, independently of variations in the number of new infections produced. We therefore used biweekly changes in the cumulative cases reported by Our World Data to explore when epidemics reached their highest growth rate during the first wave in South American countries. Most countries experienced their largest increments in the number of cumulative cases between March and April 2020. In Paraguay, Venezuela and Uruguay, however, the peak increments occurred between May and June [32] (accessed on 30 May 2021).

### Appendix A.2. Under-Reporting

Previous studies have shown that there is a non-linear relationship between the percent positivity and the magnitude of under-reporting [8]. We estimated the percentage positivity as the ratio between the number of positive RT-PCR and the number of total RT-PCR for a particular day. Thus, the number of true infections can be estimated by *Y* = $q^{b}$*X* + 1, where *X* is the number of reported infections, *b* = 0.5 and *q* is the positivity fraction [8].

### Appendix A.3. Model Description

We adapted a metapopulation SEI model developed by Li et al. [10] to estimate the trajectories of susceptible, $S_{i}$, exposed, $E_{i}$, documented infections, $I_{i}$, undocumented infections, $U_{i}$, and total population $N_{i}$ in state *i* according to,

(A1) $$\frac{dS_{i}}{dt}=-\frac{\beta S_{i}\left(I_{i}+U_{i}\right)}{N_{i}}+\theta \sum_{{}_{j}}\frac{M_{ij}S_{j}}{N_{j}-I_{j}}-\theta \sum_{j}\frac{M_{ji}S_{i}}{N_{i}-I_{i}},$$

(A2) $$\frac{dE_{i}}{dt}=\frac{\beta S_{i}\left(I_{i}+U_{i}\right)}{N_{i}}-\frac{E_{i}}{Z}+\theta \sum_{{}_{j}}\frac{M_{ij}E_{j}}{N_{j}-I_{j}}-\theta \sum_{j}\frac{M_{ji}E_{i}}{N_{i}-I_{i}},$$

(A3) $$\frac{dI_{i}}{dt}=\alpha \frac{Ei}{Z}-\frac{I_{i}}{D},$$

(A4) $$\frac{dU_{i}}{dt}=\left(1-\alpha \right)\frac{E_{i}}{Z}-\frac{U_{i}}{D}+\theta \sum_{{}_{j}}\frac{M_{ij}U_{j}}{N_{j}-I_{j}}-\theta \sum_{j}\frac{M_{ji}U_{i}}{N_{i}-I_{i}},$$

(A5) $$N_{i}=N_{i}+\theta \sum_{{}_{j}}M_{ij}-\theta \sum_{{}_{j}}M_{ji},$$

where β is the transmission rate, *Z* is the latency period, *D* the infectious period and α is the reporting rate. For movement between states within Venezuela (i,j=1,2,…,n−2), θ is the maximum number daily travellers between any two states and $M_{ij}$ is the daily number of travellers from state *i* to state *j* scaled to [0, 1] according to $M_{ij}=G_{ij}/\max_{\left\{i,j=0,1,\ldots ,n-2\right\}}G_{ij}$, with $G_{ij}$ defined by the gravity function,

(A6) $$G_{ij}=\frac{N_{i}^{\epsilon }N_{j}^{\gamma }}{e^{d_{ij}}}.$$

where the parameters ϵ and γ tune the dependence with respect to source and recipient populations and $d_{ij}$ is the distance between the centroids of each state.

Movement across country borders occurred only in one direction, from Colombia and Brazil to Venezuela. For i={1,2,…,n−2} and j={n−1,n}, $M_{ji}=0$ and $M_{ij}>0$ according to Table 1 and θ was equal to 1. Only susceptible, $S_{i}$, detected infected, $I_{i}$, or undetected infected individuals, $U_{i}$, moved between states or from neighboring countries. We assumed that transmission rates were homogeneous across all states. Differential equations were solved using a 4th order Runge–Kutta algorithm.
